# Bacterial Genomics and Epidemiology

**DOI:** 10.3390/microorganisms11061428

**Published:** 2023-05-29

**Authors:** Javier Garaizar, Lorena Laorden

**Affiliations:** MikroIker Research Group, Department of Immunology, Microbiology, and Parasitology, Faculty of Pharmacy, University of the Basque Country UPV/EHU, 01006 Vitoria-Gasteiz, Spain; lorena.laorden@ehu.eus

Innovative technologies for Whole-Genome Sequencing (WGS) help to improve our understanding of the epidemiology and pathogenesis of bacterial infectious diseases and are becoming affordable for most microbiological laboratories [1]. These recent technologies could be successfully applied to bacteria isolated from multiple reservoirs and habitats, such as clinical, food, animal, or environmental fields [2]. The “One health” concept encompasses in an interdisciplinary way the interrelation of human, animal, and environmental health [3]. This Special Issue of *Microorganisms* collects research articles and a review on these fields as examples of the aid provided by new genomic techniques in the study of bacterial infection epidemiology.

Group B *Streptococcus* (GBS), or *Streptococcus agalactiae*, is a major cause of neonatal mortality. When colonizing the lower genital tract of pregnant women, GBS may cause premature and stillbirth. If transmitted to the newborn, it may result in life-threatening illnesses, including sepsis, meningitis, and pneumonia. Moreover, through continuous evolution, GBS can use its original structure and unique factors to improve its survival rate in the human body. The review by Liu and Liu [4] discusses the key virulence factors determined by WGS that facilitate GBS invasion and colonization and their related action mechanisms. A comprehensive understanding of the role of virulence factors in GBS infection is crucial to develop better treatment options and screen potential candidate molecules for the development of a vaccine. In this sense, Shabayek et al. [5] present genomic data from a collection of colonizing GBS strains from Ismailia, Egypt, that were sequenced and characterized within the global JUNO project, resulting in a considerable proportion of serotype VI ST14 strains, a serotype which is rarely found in strain collections from the US and Europe and typically not included in current vaccine formulations. The molecular genomic epidemiology of these strains clearly points to the African origin with the detection of several sequence types that have only been observed in Africa. Their data underline the importance of the continuous molecular surveillance of the GBS population for future vaccine implementations.

*Salmonella enterica* is a leading cause of epidemic human gastrointestinal disease worldwide. Given its persistence in aquatic environments, González-López et al. [6] examined the prevalence, levels, and genotypic diversity of *Salmonella* isolates recovered from major rivers in an important agricultural region in northwestern Mexico. During a 13-month period, a total of 143 river water samples were collected and subjected to size-exclusion ultrafiltration, followed by enrichment, and selective media for the isolation and quantitation of *Salmonella*. The recovered isolates were examined by next-generation sequencing for genome characterization. Molecular subtyping revealed that Orangeburg, Anatum, and Saintpaul were the most predominant *Salmonella* serovars. Single-nucleotide polymorphism (SNP)-based phylogeny revealed that the 27 distinct serovars detected in river water clustered in two major clades. Multiple nonsynonymous SNPs were detected in *stiA*, *sivH*, and *ratA* genes required for *Salmonella*’s fitness and survival, and these findings identified relevant markers to potentially develop improved methods for characterizing this pathogen.

Finally, despite the innumerable advantages of using WGS, several drawbacks concerning data analysis, data management and exchange, and the lack of validation, hinder its integration into routine use in national reference centers or laboratories. In small or less-developed countries, the integration of WGS is particularly troublesome, as they do not always have equal access to resources compared to public health agencies or large laboratories. Data analysis also represents a bottleneck, and is becoming a serious hurdle to overcome as it typically consists of a stepwise process that is complex and tedious for non-experts. Atxaerandio-Landa et al. [7] propose a bioinformatics workflow for (Illumina) WGS data for bacterial characterization, including genome annotation, species identification, serotype prediction, antimicrobial resistance prediction, virulence-related gene and plasmid replicon detection, and core-genome-based or single nucleotide polymorphism (SNP)-based phylogenetic clustering and sequence typing. This bioinformatics workflow can be tailored to other pathogens of interest, and is freely available for academic and non-profit use as it involves an uploadable file to the Galaxy platform that does not require the use of complex informatic commands.
